# Association of erythropoietin gene polymorphism (rs1617640 C>T/G) with diabetic retinopathy in Type 2 diabetes mellitus patients of Punjabi population in Pakistan

**DOI:** 10.1371/journal.pone.0336014

**Published:** 2025-11-06

**Authors:** Cyrollah Disoma, Aqsa Malik, Ayesha Nisar, Inam Ullah, Sikandar Hayat, Abdul Qadeer, Sardar Azhar Mehmood, Sawar Khan, Zanxian Xia

**Affiliations:** 1 Department of Cell Biology, School of Life Sciences, Central South University, Changsha, China; 2 Institute of Molecular Biology and Biotechnology, The University of Lahore, Lahore, Pakistan; 3 Key Laboratory of Genetic Evolution & Animal Models, Kunming Institute of Zoology, Chinese Academy of Sciences, Kunming, Yunnan, China; 4 Department of Zoology, Hazara University, Mansehra, Pakistan; University of Tennessee Health Science Center College of Medicine Memphis, UNITED STATES OF AMERICA

## Abstract

Diabetic retinopathy (DR) is a leading cause of vision loss in individuals with type 2 diabetes mellitus (T2DM) and is influenced by genetic factors. We investigated the association between the erythropoietin (EPO) gene polymorphism (rs1617640 C > T/G) and DR risk in a Punjabi Pakistani cohort of adults with T2DM. In a case–control design, 573 T2DM patients (294 with DR, 279 controls without retinopathy, CDR) were genotyped by ARMS-PCR. The protective CC genotype was significantly more frequent in controls (96.77%) than in cases (30.61%) and was associated with markedly reduced DR risk (OR = 68; 95% CI 33.468–138.162; p < 0.001). Conversely, the GG and TT genotypes were absent in controls but present in DR patients (27.55% and 21.43%, respectively) and were strongly associated with increased DR risk (p < 0.001). Allele-level analysis mirrored genotype findings: the C allele predominated in controls (98.39%) but was significantly less frequent in cases (39%), while G and T alleles were enriched among DR patients (p < 0.001). Together, genotype- and allele-based results indicate that the rs1617640 C variant is protective against DR in this Punjabi T2DM population, whereas G and T variants increase DR susceptibility.

## Introduction

Diabetic retinopathy (DR) is one of the most severe microvascular complications associated with diabetes mellitus, and it is a leading cause of blindness worldwide, particularly among working-age adults [1–3]. The global burden of DR is escalating alongside the increasing prevalence of T2DM, particularly in low- and middle-income countries like Pakistan. In 2020, the global prevalence of DR was estimated at 103 million, with projections indicating a rise to 161 million by 2045 [4]. This alarming trend is largely driven by the rapidly growing diabetic population [5], especially in regions such as Africa, the Middle East, North Africa, and the Western Pacific. The prevalence of DR is notably high in Africa (35.90%), North America and the Caribbean (33.30%), and the Middle East and North Africa (32.90%), with significant rates also reported in the Western Pacific (19.20%), Europe (18.75%), South East Asia (16.99%), and South and Central America (13.37%) [4]. Among Asian patients with T2DM, the prevalence of DR is estimated to be around 28% [6–8]. Despite advances in T2DM management, the incidence of DR continues to rise, emphasizing the urgent need for a deeper understanding of the factors contributing to its development and progression. The pathogenesis of DR is multifactorial, involving chronic hyperglycemia, hypertension, dyslipidemia, and genetic predisposition [9]. While the roles of glycemic control and systemic factors are well-documented, the genetic component of DR is less understood. Recent studies have increasingly focused on identifying genetic markers that may contribute to the susceptibility to DR, with the aim of developing targeted prevention and treatment strategies [10–17].

Erythropoietin (EPO) is a glycoprotein hormone that plays a critical role in stimulating erythropoiesis, the process of producing red blood cells, particularly under hypoxic conditions [18]. The activation of the EPO gene occurs when hypoxia-inducible factors (HIF-1) bind to its promoter region, leading to increased EPO production [19]. Beyond its primary role in erythropoiesis, EPO has been recognized as a potent angiogenic factor, contributing to the formation of new blood vessels [20,21]. This dual function of EPO in both erythropoiesis and angiogenesis implicates it in various physiological and pathological processes, including retinal health, where its angiogenic properties may influence conditions such as DR. The DR is characterized by damage to the retinal blood vessels, primarily due to chronic hyperglycemia-induced retinal hypoxia, which activates hypoxia-inducible factors that upregulate EPO and vascular endothelial growth factor (VEGF) [22].

The rs1617640 polymorphism in the promoter region of the EPO gene has garnered significant attention due to its impact on EPO expression and its potential association with microvascular complications of DR. The T allele of rs1617640 has been linked to higher levels of EPO in the vitreous body [23], suggesting its role in DR pathogenesis, particularly in promoting retinal neovascularization in proliferative diabetic retinopathy [24]. Although some studies have found significant associations between rs1617640 and DR [22,23,25–27], others have reported no significant link [28–30]. Specifically, the T allele was associated with the risk of DR in European-Americans [23] and North Indians [25] with T2DM. These mixed findings underscore the need for further research, particularly in diverse populations, to clarify the genetic factors contributing to DR.

In Pakistan, where the prevalence of T2DM is high [31] and the population is genetically diverse [32–42], understanding the genetic risk factors for DR is of particular importance. The Punjabi population, in particular, represents a unique genetic pool with potential variations in DR susceptibility that have not been thoroughly investigated. This study was aimed to explore the association between the EPO gene polymorphism (rs1617640 C > T/G) and the risk of developing DR in Punjabi patients with T2DM.

## Methods

### Participants

This case-control study included 573 Punjabi adults with T2DM recruited from hospitals in Sheikhupura and Lahore, Pakistan, between March 2023 and May 2024. The participants were categorized into two groups: the diabetic retinopathy (DR) group, consisting of 294 subjects with T2DM and retinopathy, and the control group (CDR), consisting of 279 subjects with T2DM but without retinopathy. The diagnosis of T2DM was based on clinical features and guidelines outlined by the Expert Committee Report of the American Diabetes Association. Retinopathy status was graded according to the criteria established by the Early Treatment Diabetic Retinopathy Study [43–46]. Inclusion criteria for the DR group included individuals with retinopathy as the primary condition, diagnosed with diabetes at age 30 or older, and having an HbA1c level of 6.5% or higher. For the CDR group, participants were included if they had fasting plasma glucose levels of 150 mg/dL or higher, a duration of T2DM of at least five years, were diagnosed with diabetes at age 30 or older, and exhibited no microvascular or macrovascular complications. Individuals with type 1 diabetes or other ocular conditions such as optic neuropathy, dense cataract, or glaucoma were excluded from the study. HbA1c levels and blood urea nitrogen were measured from blood samples of all participants. Serum cholesterol, triglycerides, high-density lipoprotein (HDL) cholesterol, and low-density lipoprotein (LDL) cholesterol levels were assessed using standard kits (Cell Biolabs, Inc.). Additionally, body mass index (BMI) and blood pressure were recorded. The study protocol received approval from the Ethical review committee of Institute of Molecular Biology and Biotechnology, the University of Lahore (via letter No., Ref.IMBB/BBBC/22/230, dated December 15, 2022), and complied with the principles of the Declaration of Helsinki. All participants of the study were properly informed about the purpose of the study and were asked to complete the designed questionnaire. A written informed consent was obtained, and the objective of the research study and method of blood sampling were explained to all the participating individuals in detail.

### Inclusivity in global research

Additional information regarding the ethical, cultural, and scientific considerations specific to inclusivity in global research is included in the Supporting Information (S1 Checklist)

### Sampling, DNA extraction, and genotyping

**Fig 1:**
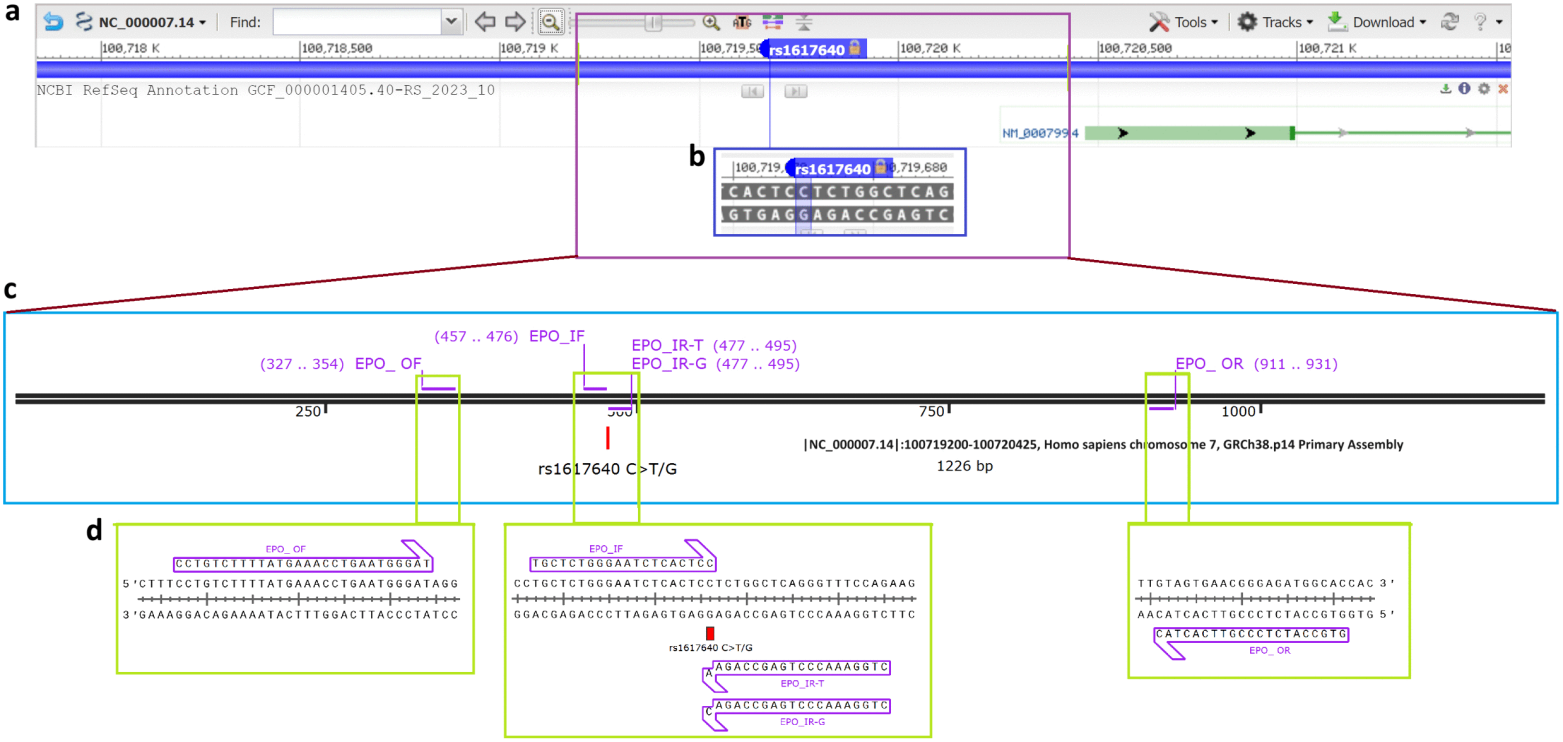
Design of tetra primers for ARMS-PCR to target the rs1617640 C > T/G polymorphism. **(a)** The target locus as visualized in the NCBI-dbSNP database. **(b)** The rs1617640polymorphism is depicted at single nucleotide resolution. **(c)** The sequence of the targeted region, including the SNP and flanking sequences (approximately 400-600 bp upstream and downstream), was downloaded for primer designing. **(d)** The designed primers are displayed at single nucleotide resolution.

Peripheral venous blood (10 mL) was collected from each participant, and genomic DNA was extracted using the phenol-chloroform extraction method (S1 File). Genotyping of the polymorphisms was performed using a polymerase chain reaction (PCR) followed by DNA sequencing. The target gene was amplified using ARMS-PCR (Amplification-Refractory Mutation System-Polymerase Chain Reaction) for subsequent analysis. The specific target sequence was retrieved from the NCBI-dbSNP database (https://www.ncbi.nlm.nih.gov/snp/) using the SNP ID rs1617640. Tetra primers for ARMS-PCR were designed to amplify the target gene fragments (Fig 1). Primer design was carried out using SnapGene software (https://www.snapgene.com/), and the primers (Table 1) were synthesized by Macrogen, South Korea, at a scale of 50 nM. A stock solution at a concentration of 100 pmol/µL and a working solution of 10 pmol/µL were prepared and stored at −20°C.

**Table 1:** Details of primers used in the present study.

Locus	Allele	Primer	Sequence (5´-3´)	GC%	Tm	Amplicon size
EPO rs1617640 C > T/G	T	EPO_OF	CCTGTCTTTTATGAAACCTGAATGGGAT	39%	59°C	169 bp
		EPO_IR-T	CTGGAAACCCTGAGCCAGAA	55%	58°C	
	G	–			169 bp	
		EPO_IR-G	CTGGAAACCCTGAGCCAGAC	60%	58°C	
	C	EPO_IF	TGCTCTGGGAATCTCACTCC	55%	58°C	475 bp
		EPO_OR	GTGCCATCTCCCGTTCACTAC	57%	59°C	

For each PCR reaction, 1 µL of genomic DNA (50 ng) was used as the template in a 25 µL reaction mixture, which included 0.125 µL (0.63 units) of Taq polymerase, 2 µL of 2 mM dNTPs, 0.7 µL of each outer primer, 0.3 µL of each inner primer, 2 µL of MgCl₂, 2.5 µL of 10X Taq buffer, and 15.375 µL of distilled water. The PCR amplification protocol included an initial denaturation at 95°C for five minutes, followed by 35 cycles of denaturation at 94°C for 30 seconds, annealing at 54°C for one minute, and extension at 72°C for 50 seconds. A final extension was performed at 72°C for ten minutes, followed by a holding step at 4°C. The PCR products were then analyzed on a 1% agarose/TAE gel under UV illumination to determine the ARMS-PCR banding patterns and genotypes. The observed band sizes were 169 bp for the mutant alleles and 475 bp for the wild-type allele corresponding to the rs1617640 C > T/G polymorphism. Allelic data were compiled in an MS Excel spreadsheet.

For further validation, the PCR products were sent to Celemics, Inc., South Korea, for commercial DNA sequencing using the BTSeq (Barcode-Tagged Sequencing) platform. The resulting sequences were analyzed for targeted peaks using SnapGene software. Multiple sequence alignments (MSA) were generated using CLUSTAL-W, as implemented in BioEdit software [47], to visualize the rs1617640 C > T/G polymorphism in the aligned sequences.

### Statistical analysis

Statistical analyses were performed using SPSS version 20 (SPSS Inc., Chicago, IL, USA). The significance of allelic frequency differences was assessed using the Chi-square test, with p-values calculated to determine statistical significance. Odds ratios (ORs) and 95% confidence intervals (CIs) were computed to evaluate the strength of the associations. A p-value of less than 0.05 was considered statistically significant. Data were presented as mean ± standard deviation (SD).

## Results

### Demographic and clinical characteristics of the sampling

The demographic and clinical characteristics of the study subjects in both DR and CDR groups were analyzed and compared (Table 2). Among the participants, the DR group included 114 males and 180 females, while the control group comprised 149 males and 130 females. Key findings from this analysis revealed significant differences between the DR and CDR groups in terms of the duration of T2DM, HbA1c levels, fasting blood glucose (FBG), total cholesterol (TC), triglycerides (TG), low-density lipoprotein (LDL), and systolic blood pressure (SBP). Specifically, the DR group had a longer duration of T2DM (p < 0.001), higher HbA1c levels (p = 0.0054), and elevated SBP (p < 0.001) compared to the control group. In contrast, the control group exhibited significantly higher levels of total cholesterol, triglycerides, and LDL (p < 0.001 for all), suggesting potential differences in lipid profiles between the groups. These clinical differences underscore the complex interplay of factors contributing to the development and progression of diabetic retinopathy. No significant difference was observed in diastolic blood pressure (DBP) between the two groups (p = 0.116), and the body mass index (BMI) was similar between both groups (p = 0.56), suggesting that these factors may not be as strongly associated with the presence of retinopathy in this cohort.

**Table 2:** Comparative analyses of the clinical parameters between diabetic retinopathy (DR) cases and control group (CDR).

Characteristics	DR (n = 294)	CDR(n = 279)	*p* Value
Age (years)	51.6 ± 12	52.1 ± 13	0.642
Duration of DM (years)	11.7 ± 6.26	7.8 ± 1.9	<0.001
HbA1c (%)	8.9 ± 2.14	8.3 ± 2.4	0.0054
BMI (kg/m^2^)	25.9 ± 3.9	26.14 ± 4.4	0.56
FBG (mg/dL)	180.78 ± 31.36	187 ± 33.2	0.021
TC (mg/dL)	170 ± 37.9	186.9 ± 48.1	<0.001
TG (mg/dL)	155.73 ± 72.42	208.3 ± 71.04	<0.001
HDL (mg/dL)	42.95 ± 21.35	40.58 ± 20.03	0.190
LDL (mg/dL)	95.26 ± 40.13	124.76 ± 15.72	<0.001
SBP (mm Hg)	134 ± 14	125 ± 20	<0.001
DBP (mm Hg)	83 ± 09	82 ± 08	0.116

Abbreviations: BMI, body mass index; CDR, controls without diabetic retinopathy; DBP, diastolic blood pressure; DR, diabetic retinopathy; FBG, fasting blood glucose; HbA1c, hemoglobin A1c; HDL, high-density lipoprotein; LDL, low-density lipoprotein; *n*, number of subjects; SBP, systolic blood pressure; TC, total cholesterol; TG, triacylglycerol.

Data expressed as mean±standard deviation

### Differential distribution of genotypes and alleles between DR and CDR

**Fig 2:**
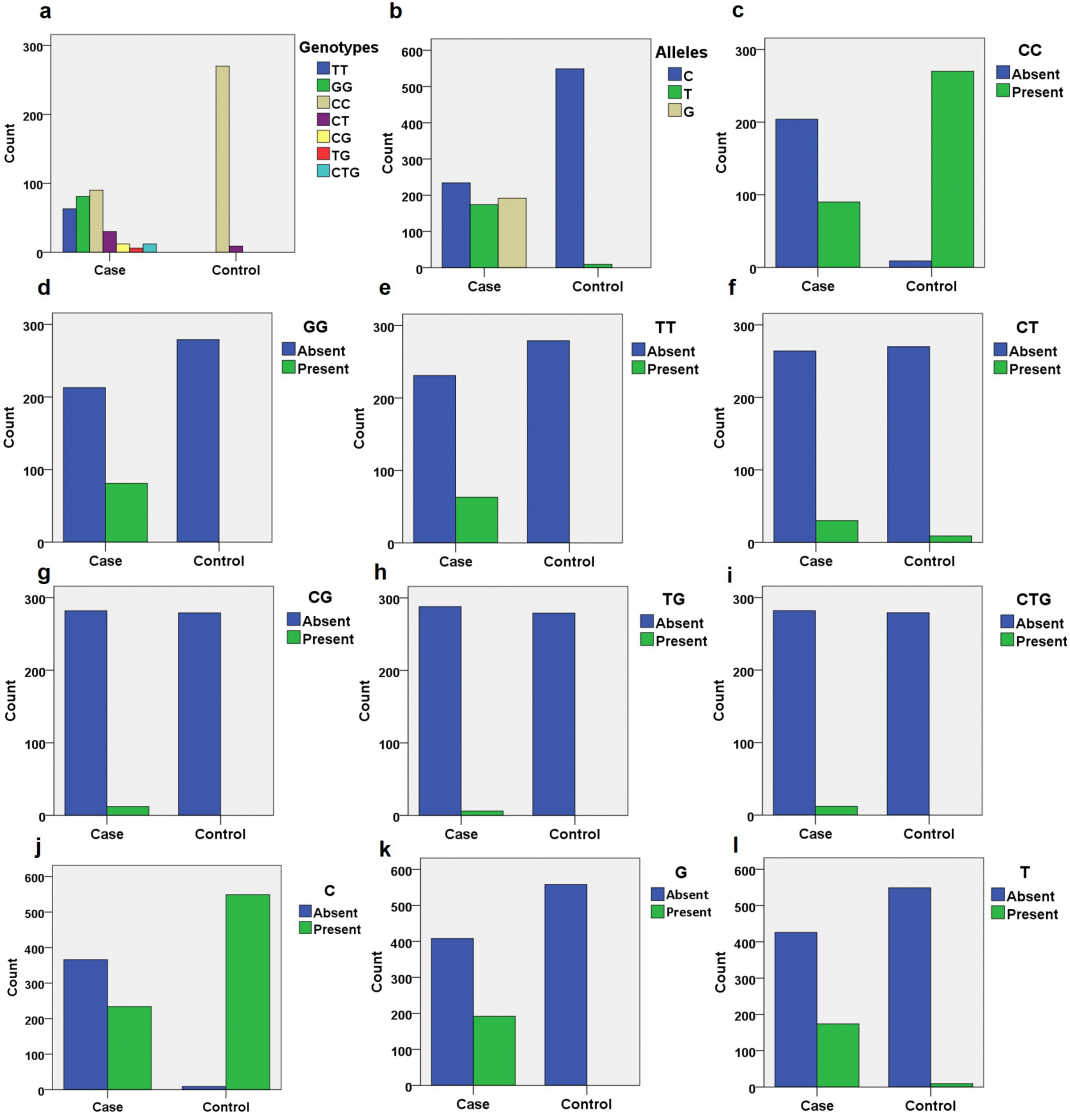
Distribution of genotypes and alleles among the case and control samples during the present study. **(a)** Overall distribution of all genotypes. **(b)** Overall distribution of all alleles. **(c)** Distribution of normal genotype CC across the groups. **(d)** Distribution of homozygous mutant genotype GG. **(e)** Distribution of homozygous mutant genotype TT. **(f)** Distribution of heterozygous mutant genotype CT. **(g)** Distribution of heterozygous mutant genotype CG. **(h)** Distribution of double mutant genotype TG. **(i)** Distribution of trisomy genotype CTG. **(j)** Distribution of normal allele C across the groups. **(k)** Distribution of mutant allele G. **(l)** Distribution of mutant allele T.

To examine the distribution of genotypes and alleles between the DR and CDR groups, we first visualized the genotypic and allelic patterns. The results indicated that genotypes and alleles were not uniformly distributed between the case and control groups (Fig 2a-b). The normal CC genotype was predominantly found in the control group (Fig 2c) but was also present in the case group. In contrast, the case group showed a higher frequency of mutant genotypes, particularly the homozygous GG and TT combinations (Fig 2d-e), compared to the heterozygous CT and CG combinations (Fig 2f-g) or the double mutant TG combination (Fig 2h). A minor presence of the trisomy genotype CTG was also observed in the case group (Fig 2i). Regarding allelic distribution, the normal C allele was predominantly observed in the control group (Fig 2j) and was also present in the case group. The mutant alleles G and T were more abundant in the case group (Fig 2k-l). Notably, the distribution of the G allele was restricted to the case group, while the T allele was predominantly observed in the case group with a minor presence in the control group (Fig 2l). These findings suggest a differential distribution of genotypes and alleles between the case and control groups, with a pronounced presence of mutant genotypes and alleles in the case group. The observed differential distribution of genotypes and alleles led for the subsequent association analysis.

### Association of rs1617640 C > T/G polymorphism with diabetic retinopathy

The analysis of genotype and allele frequencies for the EPO polymorphism (rs1617640 C > T/G) between the CDR and DR groups reveals significant associations with the occurrence of diabetic retinopathy (Table 3). The genotype distribution shows a stark contrast between the two groups. In the CDR group, the CC genotype was overwhelmingly prevalent, observed in 96.77% of individuals, while it was significantly less frequent in the DR group, where only 30.61% of participants exhibited this genotype. This substantial difference is reflected in the odds ratio of 68, with a 95% confidence interval (CI) of 33.468–138.162, and a highly significant p-value of less than 0.001, indicating a strong protective effect of the CC genotype against the development of DR.

**Table 3:** Genotype and allele frequencies for EPO polymorphism (rs1617640C>T/G) across the samples and their association with diabetic retinopathy during present study.

EPO locus	Genotype/allele	CDR (n = 279); n(%)	DR (n = 294); n(%)	Odds ratio	95% CI	p-value
rs1617640C>T/G	**Genotype**					
	CC	270 (96.77%)	90 (30.61%)	68	33.468-138.162	<0.001
	GG	0 (0%)	81 (27.55%)	0.724	0.675-0.777	<0.001
	TT	0 (0%)	63 (21.43%)	0.786	0.74-0.834	<0.001
	CT	9 (3.23%)	30 (10.20%)	0.293	0.137-0.631	0.001
	CG	0 (0%)	12 (4.08%)	0.959	0.937-0.982	<0.001
	TG	0 (0%)	6 (2.04%)	0.98	0.964-0.996	0.18
	CTG	0 (0%)	12 (4.08%)	0.959	0.937-0.982	<0.001
	**Allele**	n = 558	n = 600			
	C	549 (98.39%)	234 (39%)	95.41	48.396-188.098	<0.001
	G	0 (0%)	192 (32%)	0.68	0.644-0.718	<0.001
	T	9 (1.61%)	174 (29%)	0.04	0.02-0.079	<0.001

Conversely, the GG and TT genotypes, which were completely absent in the CDR group, were relatively common in the DR group, observed in 27.55% and 21.43% of individuals, respectively. The presence of these genotypes in the DR group is associated with significantly lower odds ratios (0.724 for GG and 0.786 for TT), both with p-values less than 0.001, suggesting a strong association between these genotypes and an increased risk of developing diabetic retinopathy. Additionally, the heterozygous CT genotype was more prevalent in the DR group (10.20%) compared to the CDR group (3.23%), with an odds ratio of 0.293 and a p-value of 0.001, further indicating its potential role in susceptibility to DR.

The allele frequency analysis further supports these findings. The C allele, which was nearly universal in the CDR group (98.39%), was significantly less frequent in the DR group (39%), corresponding to an odds ratio of 95.41 and a highly significant p-value of less than 0.001. In contrast, the G and T alleles, absent or rare in the CDR group (0% and 1.61%, respectively), were much more common in the DR group (32% for G and 29% for T). These alleles showed significantly lower odds ratios (0.68 for G and 0.04 for T) with p-values less than 0.001, indicating a strong association with diabetic retinopathy.

In sum, the data demonstrate a significant differential distribution of EPO polymorphism genotypes and alleles between the control and diabetic retinopathy groups. The CC genotype and C allele appear to be protective against diabetic retinopathy, while the GG, TT genotypes, and G and T alleles are strongly associated with an increased risk of developing this condition. These findings suggest that specific genetic variations in the EPO locus may play a critical role in the susceptibility to diabetic retinopathy.

## Discussion

This study provides a detailed analysis of the association between EPO gene polymorphism (rs1617640 C > T/G) and the risk of DR in a cohort of Punjabi patients with T2DM. The findings align with existing literature, highlighting the role of genetic variations in the EPO locus in influencing DR susceptibility, a major complication of diabetes [48].

Our results demonstrate a pronounced difference in the distribution of genotypes and alleles between the DR and CDR groups. The protective role of the CC genotype, overwhelmingly prevalent in the control group, mirrors findings from studies such as one conducted on Australian descendants, which identified a significant association between the GG genotype and increased DR risk [22]. Similarly, there is evidence suggesting a 1.6-fold increased risk of proliferative diabetic retinopathy (PDR) in individuals with the GG genotype [27], underscoring the susceptibility associated with this variant. However, our study differs in that the GG and TT genotypes were entirely absent in the control group, suggesting a stronger protective effect of the CC genotype in our Punjabi cohort, with an odds ratio of 68. Mechanistically, rs1617640 lies in the EPO promoter and has been shown to alter promoter activity and EPO expression in an allele-dependent manner [23,49]. Because erythropoietin is a potent retinal angiogenic and pro-survival factor implicated in proliferative diabetic retinopathy, a genotype/allele associated with lower EPO expression (the CC variant in our cohort) plausibly confers protection against pathological retinal neovascularization, whereas alleles that increase EPO expression promote DR — a point supported by existing genetic and meta-analytic studies [20,50].

The GG and TT genotypes, relatively common in the DR group, were associated with lower odds ratios (0.724 for GG and 0.786 for TT), indicating these genotypes may predispose individuals to DR. This observation aligns with previous research identifying the T allele of rs1617640 as a risk factor for PDR and end-stage renal disease (ESRD) in European-Americans [23]. Notably, the rs1617640, rs507392, and rs551238 polymorphisms in the EPO gene were first investigated regarding their potential association with PDR and ESRD in European-Americans, where the T allele was shown to play a functional role in EPO expression [23]. Since the initial work [23], the relationship between these genetic variants and DR has been evaluated in various populations, with mixed results. Some studies have reported positive associations with the G or T alleles, as seen in research on Australian descendants, Han Chinese, and North Indians [22,25,51]. For instance, the T allele was associated with an increased risk of DR in North Indians with T2DM [25], supporting the notion that this allele may be a risk factor in certain ethnic groups, as also suggested by our study where the C allele showed a protective effect. Conversely, other studies have found no significant association between these alleles and DR, as observed in research conducted on South Indians, Iranians, and Italians [28–30]. This disparity in findings across different populations suggests that the impact of these polymorphisms on DR risk may be influenced by ethnic and genetic backgrounds, environmental factors, or both.

Further supporting our findings, the heterozygous CT genotype also showed a higher prevalence in the DR group, with an odds ratio of 0.293, indicating its potential role in increasing susceptibility to DR. This is consistent with earlier studies that recognized higher DR risk in GG homozygotes in Han Chinese and North Indian populations [25,51]. However, the variability in results across studies, as noted in a meta-analysis, highlights the complexity of the genetic underpinnings of DR [52]. The meta-analysis, which included studies from diverse populations, revealed an association between the rs1617640 polymorphism and diabetic microvascular complications, but found no significant link between the polymorphism and DR risk in Asians or Australians.

The allele frequency analysis in our study revealed that the C allele was significantly more common in the control group, while the G and T alleles were more prevalent in the DR group. The nearly universal presence of the C allele in the control group (98.39%) and its much lower frequency in the DR group (39%) underscore its protective effect. This observation is consistent with the protective role of the C allele, as previously reported in European-Americans [23], and contrasts with the T allele’s effect observed in other populations. The differential distribution of these genotypes and alleles suggests that the EPO gene polymorphism plays a critical role in the pathogenesis of DR. The protective effect of the CC genotype and C allele, coupled with the increased risk associated with the GG, TT genotypes, and G and T alleles, provides valuable insights into the genetic mechanisms underlying DR. These findings contribute to the growing body of literature emphasizing the importance of genetic screening in T2DM patients, particularly those with a family history of DR, to identify individuals who may benefit from more aggressive management of their glycemic control and other risk factors.

However, as with previous studies, our research is not without limitations. The case-control design, while effective for identifying associations, does not establish causality. Further longitudinal studies are needed to confirm the causal relationship between EPO polymorphism and DR development. Additionally, while our study focused on a Punjabi population, the findings may not be generalizable to other ethnic groups. Future research should explore the role of EPO polymorphisms in diverse populations to better understand the global relevance of these genetic markers. The observed differences in genotype distribution between the DR and CDR groups may also be influenced by demographic and clinical factors such as age, diabetes duration, glycemic control, and lipid profiles. For instance, the DR group had significantly higher HbA1c levels and longer diabetes duration, both of which are known to exacerbate retinal damage. Additionally, lipid profiles, including elevated cholesterol and triglyceride levels in the CDR group, may contribute to the development and progression of DR. In this study, the control group exhibited elevated levels of total cholesterol, triglycerides, and LDL, which are commonly associated with cardiovascular diseases. Interestingly, these lipid abnormalities might also play a protective role by limiting the progression of DR. It is important to further explore how lipid metabolism interacts with genetic factors such as EPO polymorphisms to influence DR development. These heterogeneities should be considered when interpreting the findings, as they may affect the susceptibility to DR in different subgroups. The dual role of erythropoietin in retinal health presents a paradox. While its angiogenic properties may promote retinal blood vessel repair, in severe diabetic retinopathy, these effects may exacerbate pathological neovascularization. This highlights the complex nature of EPO’s influence on the retina, particularly in patients with advanced diabetes, where excessive angiogenesis may lead to proliferative diabetic retinopathy.

In conclusion, this study provides compelling evidence that the EPO gene polymorphism (rs1617640 C > T/G) is significantly associated with the risk of diabetic retinopathy in Punjabi patients with T2DM. The protective effect of the CC genotype and C allele, along with the increased risk conferred by the GG, TT genotypes, and G and T alleles, underscores the critical role of genetic factors in the pathogenesis of DR.

## Supporting information

S1 File DNA extraction and quality assessment. (DOCX)

S1 Checklist Inclusivity in global research. (DOCX)

S1 Data Supplementary data. (ZIP)
