# Supplementary material for: Association of erythropoietin gene polymorphism (rs1617640 C>T/G) with diabetic retinopathy in Type 2 diabetes mellitus patients of Punjabi population in Pakistan
Source: PLoS One. 2025 Nov 6;20(11):e0336014. doi: 10.1371/journal.pone.0336014 (PMC12591492; doi:10.1371/journal.pone.0336014)
Supplement: S1 File — (DOCX) [file pone.0336014.s001.docx]

**Additional File S1: DNA Extraction and Quality Assessment**

**Optimized Phenol-Chloroform DNA Extraction Protocol**
Genomic DNA was extracted using phenol-chloroform protocol optimized in our lab. The necessary reagents and their compositions are listed in Table S1.

**Table S1: Reagents and their chemical composition**

| **Sr. No** | **Reagent** | **Quantity** | **Chemical composition** | **pH** |
| --- | --- | --- | --- | --- |
| 1 | 1X PBS | 1 liter | NaCl (8.0 g), KCl (0.2 g), Na₂HPO₄ (1.42 g), KH₂PO₄ (0.24 g), and 1 liter ddH₂O | 7.4 |
| 2 | Lysis Solution | 100 ml | Tris (1.21 g), NaCl (0.52 g), EDTA (0.32 g), SDS (1.0 g), and 100 ml ddH₂O | 8.5 |
| 3 | Phenol-Chloroform | 100 ml | 50 ml phenol, 50 ml chloroform | 8 |
| 4 | 50X TAE Buffer | 250 ml | Tris base (60.6 g), Glacial Acetic Acid (14.275 ml), EDTA (4.653 g), and 250 ml ddH₂O | 8.3 |
| 5 | 1X TAE Buffer | 50 ml | 1 ml of 50X TAE buffer, 49 ml ddH₂O | 8.3 |
| 6 | 6X Loading Dye | 10 ml | Bromophenol Blue (0.025 g), Glycerol (7.06 ml), and 2.94 ml ddH₂O | 7 |
| 7 | Ethidium Bromide | 5 ml | Ethidium Bromide (0.5 mg) and 5 ml ddH₂O | 8 |

**DNA extraction protocol**

1. Thaw EDTA blood tubes and transfer 300 µl of blood into a pre-labeled 1.5 ml Eppendorf tube.
2. Add 300 µl of 1X PBS to each tube.
3. Add 300 µl of lysis solution to each sample and vortex for 10 seconds.
4. Add 500 µl of phenol-chloroform (1:1) to each sample and vortex until the mixture turns milky.
5. Centrifuge samples at 12,000 rpm for 15 minutes and transfer the upper (supernatant) layer to a new Eppendorf tube.
6. Add 300 µl of chloroform, centrifuge at 12,000 rpm for 10 minutes, and transfer the upper layer to a new tube.
7. Add 500-600 µl of chilled isopropanol and freeze samples at -20 °C for 30 minutes.
8. Centrifuge at 12,000 rpm for 20 minutes, discard the isopropanol, and collect the DNA pellet at the tube's bottom.
9. Add 500 µl of 75% ethanol and centrifuge at 12,000 rpm for 10 minutes.
10. Discard the ethanol and air-dry the samples at room temperature for 20 minutes.
11. Dissolve the DNA pellet in 20 µl of ddH₂O and store at -20 °C.

**Quality and quantity assessment of extracted DNA**
The quality and quantity of the extracted DNA were assessed using NanoDrop spectrophotometry and agarose gel electrophoresis.

**Agarose gel electrophoresis protocol**

1. Prepare the gel by dissolving 1 g of agarose powder in 100 ml of 1X TAE buffer, heating until completely dissolved.
2. Cool the solution to approximately 45 °C at room temperature for 15-20 minutes.
3. Add 3 µl of Ethidium Bromide, pour the solution into a gel tray, and insert the well comb.
4. Once solidified (20-25 minutes), remove the comb and place the gel tray into an electrophoresis apparatus containing 250-300 ml of 1X TAE buffer.
5. Mix 3 µl of DNA sample with 2 µl of 6X DNA loading dye and load into the wells.
6. Run the gel at 100 V for 20 minutes and visualize under UV light.
