# Supplementary material for: Association of erythropoietin gene polymorphism (rs1617640 C>T/G) with diabetic retinopathy in Type 2 diabetes mellitus patients of Punjabi population in Pakistan
Source: PLoS One. 2025 Nov 6;20(11):e0336014. doi: 10.1371/journal.pone.0336014 (PMC12591492; doi:10.1371/journal.pone.0336014)

**SPSS data:**

| **Groups * Genotyping Crosstabulation** | | | | | | | | | |
| --- | --- | --- | --- | --- | --- | --- | --- | --- | --- |
| Count | | | | | | | | | |
|  | | Genotyping | | | | | | | Total |
|  |  | TT | GG | CC | CT | CG | TG | CTG |  |
| Groups | Case | 63 | 81 | 90 | 30 | 12 | 6 | 12 | 294 |
|  | Control | 0 | 0 | 270 | 9 | 0 | 0 | 0 | 279 |
| Total | | 63 | 81 | 360 | 39 | 12 | 6 | 12 | 573 |

| **Chi-Square Tests** | | | |
| --- | --- | --- | --- |
|  | Value | df | Asymp. Sig. (2-sided) |
| Pearson Chi-Square | 275.104^a^ | 6 | .000 |
| Likelihood Ratio | 346.937 | 6 | .000 |
| Linear-by-Linear Association | 13.456 | 1 | .000 |
| N of Valid Cases | 573 |  |  |
| a. 2 cells (14.3%) have expected count less than 5. The minimum expected count is 2.92. | | | |

| **Risk Estimate** | |
| --- | --- |
|  | Value |
| Odds Ratio for Groups (Case / Control) | ^a^ |
| a. Risk Estimate statistics cannot be computed. They are only computed for a 2*2 table without empty cells. | |


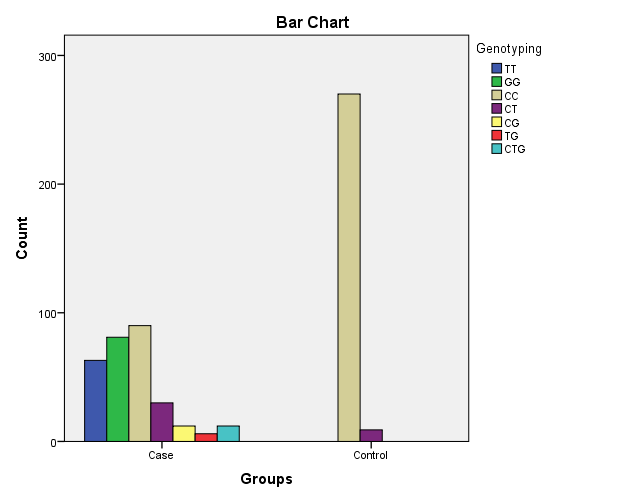


CROSSTABS

/TABLES=Groups BY TT GG CC CT CG TG CTG

/FORMAT=AVALUE TABLES

/STATISTICS=CHISQ RISK

/CELLS=COUNT

/COUNT ROUND CELL

/BARCHART.

**Crosstabs**

**Groups * TT**

| **Crosstab** | | | | |
| --- | --- | --- | --- | --- |
| Count | | | | |
|  | | TT | | Total |
|  |  | Absent | Present |  |
| Groups | Case | 231 | 63 | 294 |
|  | Control | 279 | 0 | 279 |
| Total | | 510 | 63 | 573 |

| **Chi-Square Tests** | | | | | |
| --- | --- | --- | --- | --- | --- |
|  | Value | df | Asymp. Sig. (2-sided) | Exact Sig. (2-sided) | Exact Sig. (1-sided) |
| Pearson Chi-Square | 67.171^a^ | 1 | .000 |  |  |
| Continuity Correction^b^ | 64.999 | 1 | .000 |  |  |
| Likelihood Ratio | 91.468 | 1 | .000 |  |  |
| Fisher's Exact Test |  |  |  | .000 | .000 |
| Linear-by-Linear Association | 67.054 | 1 | .000 |  |  |
| N of Valid Cases | 573 |  |  |  |  |
| a. 0 cells (0.0%) have expected count less than 5. The minimum expected count is 30.68. | | | | | |
| b. Computed only for a 2x2 table | | | | | |

| **Risk Estimate** | | | |
| --- | --- | --- | --- |
|  | Value | 95% Confidence Interval | |
|  |  | Lower | Upper |
| For cohort TT = Absent | .786 | .740 | .834 |
| N of Valid Cases | 573 |  |  |


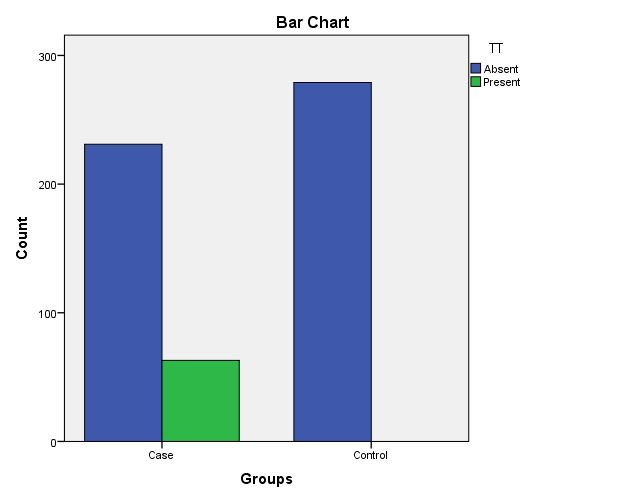


**Groups * GG**

| **Crosstab** | | | | |
| --- | --- | --- | --- | --- |
| Count | | | | |
|  | | GG | | Total |
|  |  | Absent | Present |  |
| Groups | Case | 213 | 81 | 294 |
|  | Control | 279 | 0 | 279 |
| Total | | 492 | 81 | 573 |

| **Chi-Square Tests** | | | | | |
| --- | --- | --- | --- | --- | --- |
|  | Value | df | Asymp. Sig. (2-sided) | Exact Sig. (2-sided) | Exact Sig. (1-sided) |
| Pearson Chi-Square | 89.522^a^ | 1 | .000 |  |  |
| Continuity Correction^b^ | 87.267 | 1 | .000 |  |  |
| Likelihood Ratio | 120.778 | 1 | .000 |  |  |
| Fisher's Exact Test |  |  |  | .000 | .000 |
| Linear-by-Linear Association | 89.366 | 1 | .000 |  |  |
| N of Valid Cases | 573 |  |  |  |  |
| a. 0 cells (0.0%) have expected count less than 5. The minimum expected count is 39.44. | | | | | |
| b. Computed only for a 2x2 table | | | | | |

| **Risk Estimate** | | | |
| --- | --- | --- | --- |
|  | Value | 95% Confidence Interval | |
|  |  | Lower | Upper |
| For cohort GG = Absent | .724 | .675 | .777 |
| N of Valid Cases | 573 |  |  |


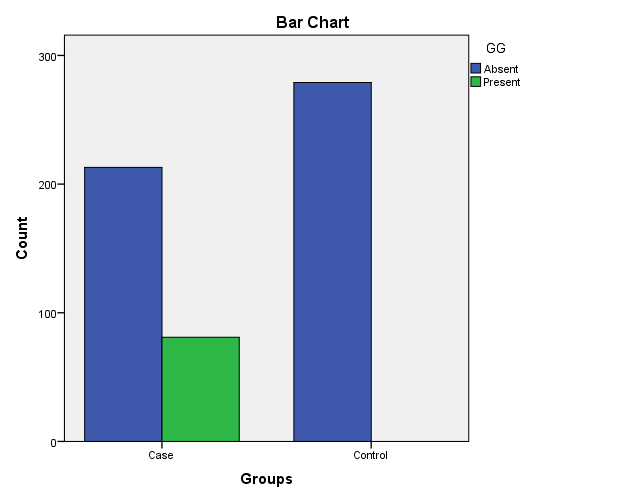


**Groups * CC**

| **Crosstab** | | | | |
| --- | --- | --- | --- | --- |
| Count | | | | |
|  | | CC | | Total |
|  |  | Absent | Present |  |
| Groups | Case | 204 | 90 | 294 |
|  | Control | 9 | 270 | 279 |
| Total | | 213 | 360 | 573 |

| **Chi-Square Tests** | | | | | |
| --- | --- | --- | --- | --- | --- |
|  | Value | df | Asymp. Sig. (2-sided) | Exact Sig. (2-sided) | Exact Sig. (1-sided) |
| Pearson Chi-Square | 268.312^a^ | 1 | .000 |  |  |
| Continuity Correction^b^ | 265.487 | 1 | .000 |  |  |
| Likelihood Ratio | 314.505 | 1 | .000 |  |  |
| Fisher's Exact Test |  |  |  | .000 | .000 |
| Linear-by-Linear Association | 267.844 | 1 | .000 |  |  |
| N of Valid Cases | 573 |  |  |  |  |
| a. 0 cells (0.0%) have expected count less than 5. The minimum expected count is 103.71. | | | | | |
| b. Computed only for a 2x2 table | | | | | |

| **Risk Estimate** | | | |
| --- | --- | --- | --- |
|  | Value | 95% Confidence Interval | |
|  |  | Lower | Upper |
| Odds Ratio for Groups (Case / Control) | 68.000 | 33.468 | 138.162 |
| For cohort CC = Absent | 21.510 | 11.261 | 41.087 |
| For cohort CC = Present | .316 | .266 | .376 |
| N of Valid Cases | 573 |  |  |


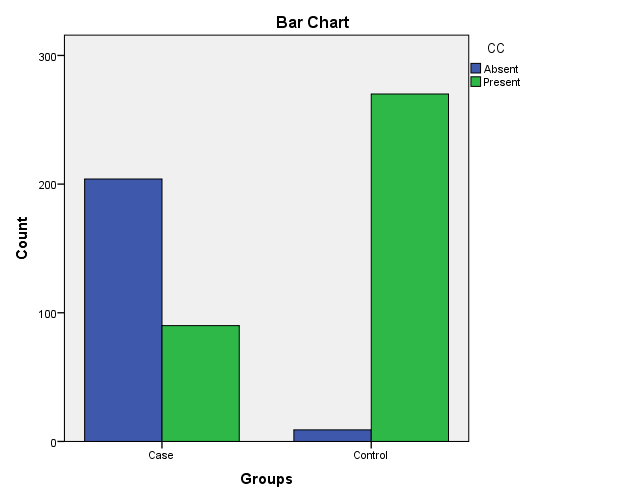


**Groups * CT**

| **Crosstab** | | | | |
| --- | --- | --- | --- | --- |
| Count | | | | |
|  | | CT | | Total |
|  |  | Absent | Present |  |
| Groups | Case | 264 | 30 | 294 |
|  | Control | 270 | 9 | 279 |
| Total | | 534 | 39 | 573 |

| **Chi-Square Tests** | | | | | |
| --- | --- | --- | --- | --- | --- |
|  | Value | df | Asymp. Sig. (2-sided) | Exact Sig. (2-sided) | Exact Sig. (1-sided) |
| Pearson Chi-Square | 10.990^a^ | 1 | .001 |  |  |
| Continuity Correction^b^ | 9.917 | 1 | .002 |  |  |
| Likelihood Ratio | 11.604 | 1 | .001 |  |  |
| Fisher's Exact Test |  |  |  | .001 | .001 |
| Linear-by-Linear Association | 10.971 | 1 | .001 |  |  |
| N of Valid Cases | 573 |  |  |  |  |
| a. 0 cells (0.0%) have expected count less than 5. The minimum expected count is 18.99. | | | | | |
| b. Computed only for a 2x2 table | | | | | |

| **Risk Estimate** | | | |
| --- | --- | --- | --- |
|  | Value | 95% Confidence Interval | |
|  |  | Lower | Upper |
| Odds Ratio for Groups (Case / Control) | .293 | .137 | .630 |
| For cohort CT = Absent | .928 | .888 | .970 |
| For cohort CT = Present | 3.163 | 1.529 | 6.542 |
| N of Valid Cases | 573 |  |  |


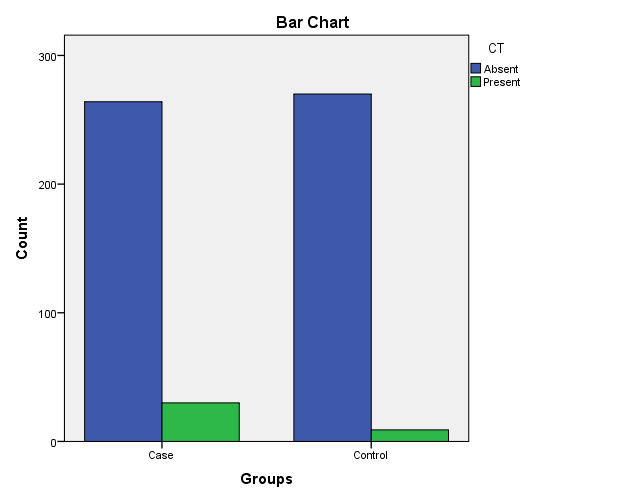


**Groups * CG**

| **Crosstab** | | | | |
| --- | --- | --- | --- | --- |
| Count | | | | |
|  | | CG | | Total |
|  |  | Absent | Present |  |
| Groups | Case | 282 | 12 | 294 |
|  | Control | 279 | 0 | 279 |
| Total | | 561 | 12 | 573 |

| **Chi-Square Tests** | | | | | |
| --- | --- | --- | --- | --- | --- |
|  | Value | df | Asymp. Sig. (2-sided) | Exact Sig. (2-sided) | Exact Sig. (1-sided) |
| Pearson Chi-Square | 11.631^a^ | 1 | .001 |  |  |
| Continuity Correction^b^ | 9.726 | 1 | .002 |  |  |
| Likelihood Ratio | 16.259 | 1 | .000 |  |  |
| Fisher's Exact Test |  |  |  | .000 | .000 |
| Linear-by-Linear Association | 11.611 | 1 | .001 |  |  |
| N of Valid Cases | 573 |  |  |  |  |
| a. 0 cells (0.0%) have expected count less than 5. The minimum expected count is 5.84. | | | | | |
| b. Computed only for a 2x2 table | | | | | |

| **Risk Estimate** | | | |
| --- | --- | --- | --- |
|  | Value | 95% Confidence Interval | |
|  |  | Lower | Upper |
| For cohort CG = Absent | .959 | .937 | .982 |
| N of Valid Cases | 573 |  |  |


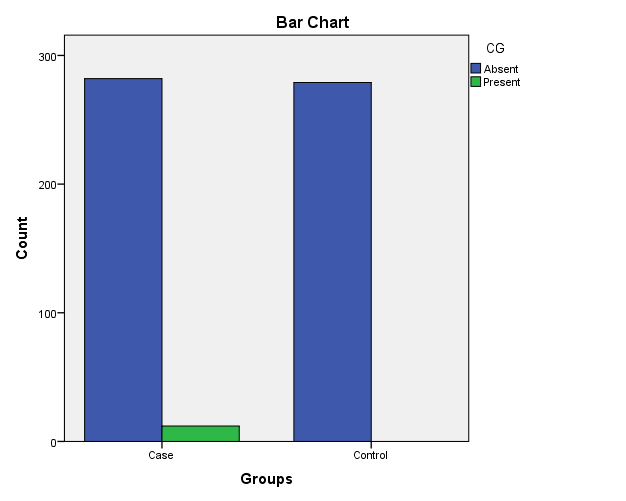


**Groups * TG**

| **Crosstab** | | | | |
| --- | --- | --- | --- | --- |
| Count | | | | |
|  | | TG | | Total |
|  |  | Absent | Present |  |
| Groups | Case | 288 | 6 | 294 |
|  | Control | 279 | 0 | 279 |
| Total | | 567 | 6 | 573 |

| **Chi-Square Tests** | | | | | |
| --- | --- | --- | --- | --- | --- |
|  | Value | df | Asymp. Sig. (2-sided) | Exact Sig. (2-sided) | Exact Sig. (1-sided) |
| Pearson Chi-Square | 5.754^a^ | 1 | .016 |  |  |
| Continuity Correction^b^ | 3.953 | 1 | .047 |  |  |
| Likelihood Ratio | 8.068 | 1 | .005 |  |  |
| Fisher's Exact Test |  |  |  | .031 | .018 |
| Linear-by-Linear Association | 5.744 | 1 | .017 |  |  |
| N of Valid Cases | 573 |  |  |  |  |
| a. 2 cells (50.0%) have expected count less than 5. The minimum expected count is 2.92. | | | | | |
| b. Computed only for a 2x2 table | | | | | |

| **Risk Estimate** | | | |
| --- | --- | --- | --- |
|  | Value | 95% Confidence Interval | |
|  |  | Lower | Upper |
| For cohort TG = Absent | .980 | .964 | .996 |
| N of Valid Cases | 573 |  |  |


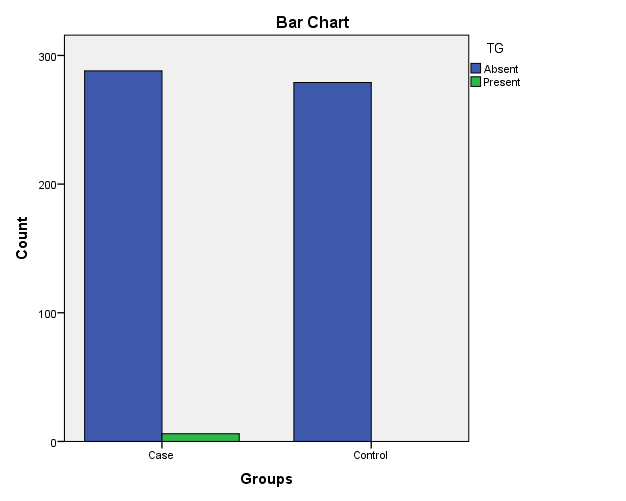


**Groups * CTG**

| **Crosstab** | | | | |
| --- | --- | --- | --- | --- |
| Count | | | | |
|  | | CTG | | Total |
|  |  | Absent | Present |  |
| Groups | Case | 282 | 12 | 294 |
|  | Control | 279 | 0 | 279 |
| Total | | 561 | 12 | 573 |

| **Chi-Square Tests** | | | | | |
| --- | --- | --- | --- | --- | --- |
|  | Value | df | Asymp. Sig. (2-sided) | Exact Sig. (2-sided) | Exact Sig. (1-sided) |
| Pearson Chi-Square | 11.631^a^ | 1 | .001 |  |  |
| Continuity Correction^b^ | 9.726 | 1 | .002 |  |  |
| Likelihood Ratio | 16.259 | 1 | .000 |  |  |
| Fisher's Exact Test |  |  |  | .000 | .000 |
| Linear-by-Linear Association | 11.611 | 1 | .001 |  |  |
| N of Valid Cases | 573 |  |  |  |  |
| a. 0 cells (0.0%) have expected count less than 5. The minimum expected count is 5.84. | | | | | |
| b. Computed only for a 2x2 table | | | | | |

| **Risk Estimate** | | | |
| --- | --- | --- | --- |
|  | Value | 95% Confidence Interval | |
|  |  | Lower | Upper |
| For cohort CTG = Absent | .959 | .937 | .982 |
| N of Valid Cases | 573 |  |  |


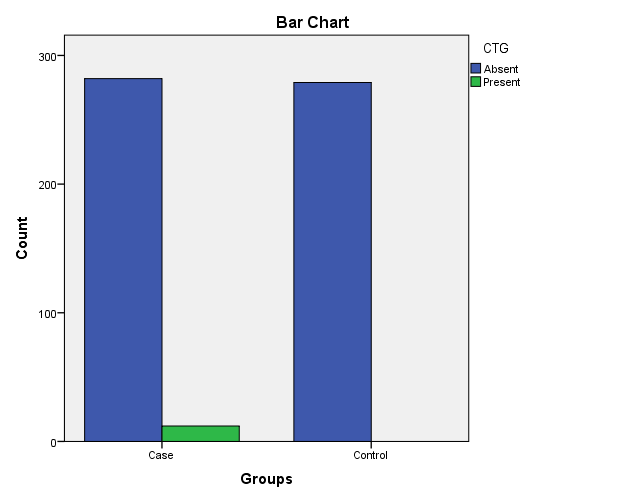


**Alleles:**

CROSSTABS

/TABLES=Groups BY Alleles

/FORMAT=AVALUE TABLES

/STATISTICS=CHISQ RISK

/CELLS=COUNT

/COUNT ROUND CELL

/BARCHART.

**Crosstabs**

| **Groups * Alleles Crosstabulation** | | | | | |
| --- | --- | --- | --- | --- | --- |
| Count | | | | | |
|  | | Alleles | | | Total |
|  |  | C | T | G |  |
| Groups | Case | 234 | 174 | 192 | 600 |
|  | Control | 549 | 9 | 0 | 558 |
| Total | | 783 | 183 | 192 | 1158 |

| **Chi-Square Tests** | | | |
| --- | --- | --- | --- |
|  | Value | df | Asymp. Sig. (2-sided) |
| Pearson Chi-Square | 466.585^a^ | 2 | .000 |
| Likelihood Ratio | 576.951 | 2 | .000 |
| Linear-by-Linear Association | 414.880 | 1 | .000 |
| N of Valid Cases | 1158 |  |  |
| a. 0 cells (0.0%) have expected count less than 5. The minimum expected count is 88.18. | | | |

| **Risk Estimate** | |
| --- | --- |
|  | Value |
| Odds Ratio for Groups (Case / Control) | ^a^ |
| a. Risk Estimate statistics cannot be computed. They are only computed for a 2*2 table without empty cells. | |


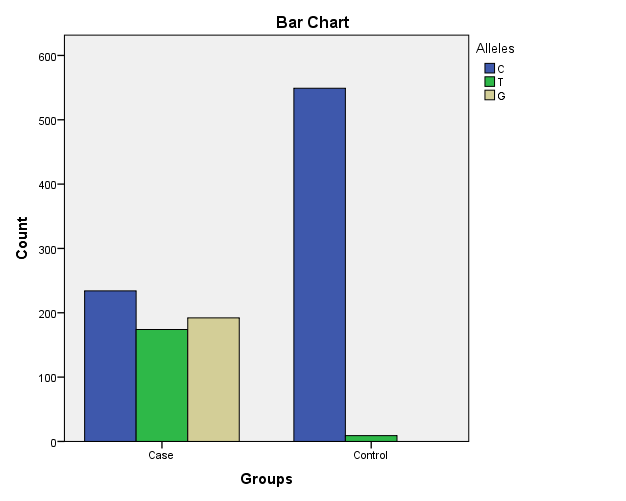


**Crosstabs**

**Groups * C**

| **Crosstab** | | | | |
| --- | --- | --- | --- | --- |
| Count | | | | |
|  | | C | | Total |
|  |  | Absent | Present |  |
| Groups | Case | 366 | 234 | 600 |
|  | Control | 9 | 549 | 558 |
| Total | | 375 | 783 | 1158 |

| **Chi-Square Tests** | | | | | |
| --- | --- | --- | --- | --- | --- |
|  | Value | df | Asymp. Sig. (2-sided) | Exact Sig. (2-sided) | Exact Sig. (1-sided) |
| Pearson Chi-Square | 465.677^a^ | 1 | .000 |  |  |
| Continuity Correction^b^ | 462.969 | 1 | .000 |  |  |
| Likelihood Ratio | 563.805 | 1 | .000 |  |  |
| Fisher's Exact Test |  |  |  | .000 | .000 |
| Linear-by-Linear Association | 465.275 | 1 | .000 |  |  |
| N of Valid Cases | 1158 |  |  |  |  |
| a. 0 cells (0.0%) have expected count less than 5. The minimum expected count is 180.70. | | | | | |
| b. Computed only for a 2x2 table | | | | | |

| **Risk Estimate** | | | |
| --- | --- | --- | --- |
|  | Value | 95% Confidence Interval | |
|  |  | Lower | Upper |
| Odds Ratio for Groups (Case / Control) | 95.410 | 48.396 | 188.098 |
| For cohort C = Absent | 37.820 | 19.720 | 72.531 |
| For cohort C = Present | .396 | .358 | .438 |
| N of Valid Cases | 1158 |  |  |


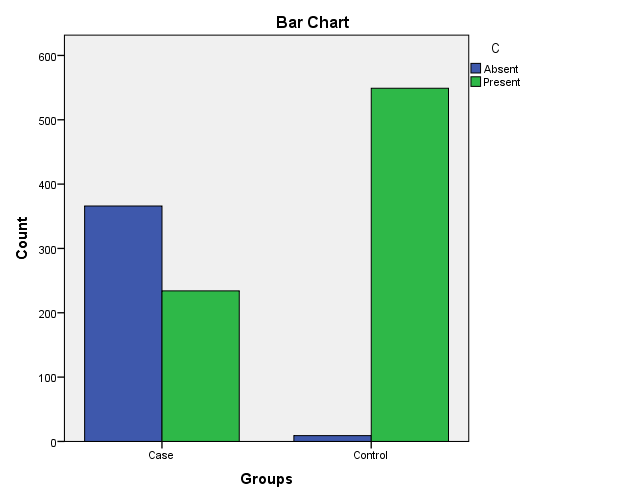


**Groups * T**

| **Crosstab** | | | | |
| --- | --- | --- | --- | --- |
| Count | | | | |
|  | | T | | Total |
|  |  | Absent | Present |  |
| Groups | Case | 426 | 174 | 600 |
|  | Control | 549 | 9 | 558 |
| Total | | 975 | 183 | 1158 |

| **Chi-Square Tests** | | | | | |
| --- | --- | --- | --- | --- | --- |
|  | Value | df | Asymp. Sig. (2-sided) | Exact Sig. (2-sided) | Exact Sig. (1-sided) |
| Pearson Chi-Square | 162.978^a^ | 1 | .000 |  |  |
| Continuity Correction^b^ | 160.927 | 1 | .000 |  |  |
| Likelihood Ratio | 195.956 | 1 | .000 |  |  |
| Fisher's Exact Test |  |  |  | .000 | .000 |
| Linear-by-Linear Association | 162.838 | 1 | .000 |  |  |
| N of Valid Cases | 1158 |  |  |  |  |
| a. 0 cells (0.0%) have expected count less than 5. The minimum expected count is 88.18. | | | | | |
| b. Computed only for a 2x2 table | | | | | |

| **Risk Estimate** | | | |
| --- | --- | --- | --- |
|  | Value | 95% Confidence Interval | |
|  |  | Lower | Upper |
| Odds Ratio for Groups (Case / Control) | .040 | .020 | .079 |
| For cohort T = Absent | .722 | .685 | .760 |
| For cohort T = Present | 17.980 | 9.293 | 34.788 |
| N of Valid Cases | 1158 |  |  |


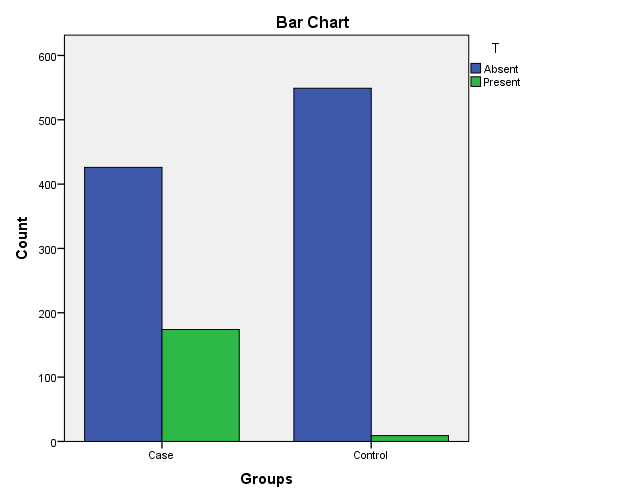


**Groups * G**

| **Crosstab** | | | | |
| --- | --- | --- | --- | --- |
| Count | | | | |
|  | | G | | Total |
|  |  | Absent | Present |  |
| Groups | Case | 408 | 192 | 600 |
|  | Control | 558 | 0 | 558 |
| Total | | 966 | 192 | 1158 |

| **Chi-Square Tests** | | | | | |
| --- | --- | --- | --- | --- | --- |
|  | Value | df | Asymp. Sig. (2-sided) | Exact Sig. (2-sided) | Exact Sig. (1-sided) |
| Pearson Chi-Square | 214.050^a^ | 1 | .000 |  |  |
| Continuity Correction^b^ | 211.743 | 1 | .000 |  |  |
| Likelihood Ratio | 288.031 | 1 | .000 |  |  |
| Fisher's Exact Test |  |  |  | .000 | .000 |
| Linear-by-Linear Association | 213.865 | 1 | .000 |  |  |
| N of Valid Cases | 1158 |  |  |  |  |
| a. 0 cells (0.0%) have expected count less than 5. The minimum expected count is 92.52. | | | | | |
| b. Computed only for a 2x2 table | | | | | |

| **Risk Estimate** | | | |
| --- | --- | --- | --- |
|  | Value | 95% Confidence Interval | |
|  |  | Lower | Upper |
| For cohort G = Absent | .680 | .644 | .718 |
| N of Valid Cases | 1158 |  |  |


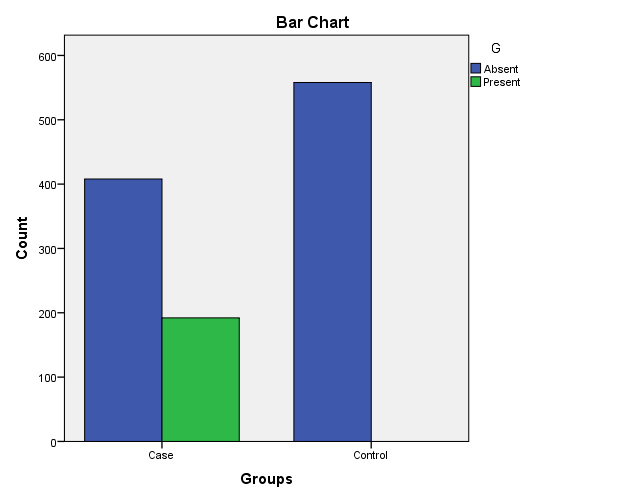

Supplement: S1 Data — (ZIP) [file pone.0336014.s003.zip › Supplementary_Data/S1 Data_Supplementary data_SPSS data.docx]
